# Modulation of three key innate immune pathways for the most common retinal degenerative diseases

**DOI:** 10.15252/emmm.201708259

**Published:** 2018-09-17

**Authors:** Isha Akhtar‐Schäfer, Luping Wang, Tim U Krohne, Heping Xu, Thomas Langmann

**Affiliations:** ^1^ Laboratory for Experimental Immunology of the Eye Department of Ophthalmology University of Cologne Cologne Germany; ^2^ Department of Ophthalmology University of Bonn Bonn Germany; ^3^ Centre for Experimental Medicine The Wellcome‐Wolfson Institute for Experimental Medicine School of Medicine, Dentistry & Biomedical Sciences Queen's University Belfast Belfast UK; ^4^ Center for Molecular Medicine University of Cologne Cologne Germany

**Keywords:** complement, inflammasome, microglia, mononuclear phagocytes, retina, Immunology, Neuroscience

## Abstract

This review highlights the role of three key immune pathways in the pathophysiology of major retinal degenerative diseases including diabetic retinopathy, age‐related macular degeneration, and rare retinal dystrophies. We first discuss the mechanisms how loss of retinal homeostasis evokes an unbalanced retinal immune reaction involving responses of local microglia and recruited macrophages, activity of the alternative complement system, and inflammasome assembly in the retinal pigment epithelium. Presenting these key mechanisms as complementary targets, we specifically emphasize the concept of immunomodulation as potential treatment strategy to prevent or delay vision loss. Promising molecules are ligands for phagocyte receptors, specific inhibitors of complement activation products, and inflammasome inhibitors. We comprehensively summarize the scientific evidence for this strategy from preclinical animal models, human ocular tissue analyses, and clinical trials evolving in the last few years.

**Glossary**
Fate‐mapping: A method applied in developmental biology, for understanding the embryonic origin of tissues in the adult organism by investigating the correspondence between individual cells (or groups of cells) at one stage of development, and their progeny at later stages.
Geographic atrophy (GA): An advanced form of AMD characterized by the presence of atrophic lesions of the outer retina, resulting from loss of photoreceptors, retinal pigment epithelium (RPE), and underlying choriocapillaris.
Humanized antibodies: Antibodies from non‐human species whose protein sequences have been modified to increase their similarity to antibody variants produced naturally in humans in order to reduce the immunogenicity.
Immunomodulation: Therapeutic interventions modulating the immune response to a desired level rather than suppressing it. The aim is to enhance beneficial functions while minimizing host harming processes of the immune system.
Innate immune system: Nonspecific defense mechanisms that deliver host defense immediately or within hours of pathogen appearance or tissue insult.
Microglia: Resident immune cells of the brain and retina that are derived from primitive myeloid progenitors originating from the yolk sac. Microglia cells are a long‐living, autonomous, and self‐renewing population and are not replenished from postnatal hematopoietic.
Mononuclear phagocytes: Mononuclear cells include circulating blood monocytes, tissue‐resident macrophages, dendritic cells, and microglia with the ability to phagocytose.
Non‐proliferative diabetic retinopathy (non‐PDR): An early stage of diabetic retinopathy characterized by damage to retinal vasculature and loss of pericytes. It can further progress into PDR defined by pathological neovascular growth, vitreous hemorrhage, retinal scars, and detachment, resulting in irreversible vision loss.
Ontogeny: All the developmental events that occur during the existence of a living organism. In cell biology, ontogeny refers specifically to developmental and differentiation processes within a cell lineage.
Phagoptosis (primary phagocytosis): Cell death resulting from phagocytosis of reversibly stressed cells by phagocytes, provoked by exposure of “eat‐me” signals (e.g., phosphatidylserine) and/or loss of “don't‐eat‐me” signals (e.g., polysialic acid).
*Rd*1 and *rd*10: Two of the 16 naturally occurring mouse mutant lines that manifest degeneration of the photoreceptors. *Rd*1 and *rd*10 mice carry mutation in exon 7 and exon 13 of the beta subunit of the rod phosphodiesterase gene, respectively.

## Introduction

Diabetic retinopathy (DR) and age‐related macular degeneration (AMD) are the two most frequent retinal degenerative and neovascular diseases in the developed world. While the former is an end‐stage diabetic complication and leading cause of visual impairment among working‐age adults, the latter is the most common cause of blindness in the elderly, especially among Caucasians. Around one‐third of the population is diagnosed with diabetes, with one‐tenth having vision‐threatening disease course which includes diabetic macular edema (DME) or proliferative diabetic retinopathy (PDR; Ting *et* *al,* 2016). Also alarming are the epidemiologic facts about AMD with more than 150 million people worldwide suffering from early forms and around 10 million people developing the late stages which are geographic atrophy (GA) and neovascular AMD (Wong *et* *al,* 2014). Characteristic for DR and the neovascular form of AMD is blood vessel growth from the subretinal space into the retina. The vascular network not only forms in an unregulated manner, but also becomes leaky. New vessel formation is driven by the angiogenic factor vascular endothelial growth factor (VEGF), and hence, both diseases are treated with intravitreal injections of VEGF inhibitors; however, treatment success is not guaranteed (Cummings & Cunha‐Vaz, 2008). Moreover, no treatment options are currently available for patients suffering from GA. Another group of blinding diseases lacking established therapeutic options are inherited retinal degenerations, such as retinitis pigmentosa (RP). RP is the most frequent monogenic photoreceptor degenerating disease with an estimated prevalence of 1:4,000 (Haim, 2002).

The vertebrate retina is a highly organized layered structure with more than 60 distinct cell types (Masland, 2001; Hoon *et* *al,* 2014). Both the highly active photoreceptor cells and the phagocytic retinal pigment epithelium cells (RPE) contribute to the generation of metabolic by‐products (Chiu & Taylor, 2011; Datta *et* *al,* 2017). With increasing age, there is a decline in functionality of retinal cells (Damani *et* *al,* 2011; Mitter *et* *al,* 2014). Hence, the cells are less effective in dealing with the accumulating metabolic waste (Wang *et* *al,* 2009; Mitter *et* *al,* 2014). Moreover, the decline in functionality is accompanied by a drop in efficacy, for instance, of the RPE to phagocyte shedded photoreceptor debris (Nandrot *et* *al,* 2004; Gu *et* *al,* 2012; Mazzoni *et* *al,* 2014). Throughout lifetime, this complex organ is challenged by a variety of noxious insults including hypoxia, hyperglycemia, and inherited mutations (Fritsche *et* *al,* 2016; Masuda *et* *al,* 2017). These circumstances demand constant surveillance of the retina for the detection and defense against pathologic perturbation. To meet this demand, the retina is equipped with a highly sensitive innate immune system. This immune system includes (i) surveilling microglia cells, which migrate to the site of damage and phagocyte apoptotic material (Karlstetter *et* *al,* 2015), (ii) activation of the complement system to opsonize cellular debris (Xu & Chen, 2016), and (iii) inflammasome assembly in the RPE (Doyle *et* *al,* 2012; Gao *et* *al,* 2015). When not tightly controlled, these immune pathways pose threat to the surrounding host tissue. Hence, a cross‐talk with Müller cells and retinal neurons through the release of regulatory molecules, including complement factors, chemokines, and neurotrophic factors, limits overt immune activation in the healthy retina (Harada *et* *al,* 2002; Langmann, 2007; Wolf *et* *al,* 2013). In the event of a transient imbalance in retinal physiology, rapid activation of the immune response will induce restoration of tissue homeostasis and function. However, in case of persistent insult, chronic over‐activation of the inflammatory response can lead to devastating tissue remodeling (Chen & Xu, 2015). Pro‐inflammatory factors such as reactive oxygen species (ROS), TNF‐α, and CCL2 as well as complement activators such as C1q are released into the cytosol by overly active microglia (Scholz *et* *al,* 2015a; Madeira *et* *al,* 2018). Furthermore, microglia over‐express the anaphylatoxin receptors C5aR and C3aR, while complement inhibitors such as CFH and CFI are downregulated (Zipfel & Skerka, 2009; Guillonneau *et* *al,* 2017; Madeira *et* *al,* 2018). Complement factors also act as enhanced triggers for inflammasome assembly, which leads to the activation of the pro‐inflammatory cytokines pro‐IL‐1β and pro‐IL‐18 (Nebel *et* *al,* 2017; Madeira *et* *al,* 2018). The resulting chronic inflammatory response is associated with a decline in RPE function and structure, breach of the blood–retina barrier (BRB), new vessel formation, and recruitment of choroidal macrophages (Donoso *et* *al,* 2006; Liu *et* *al,* 2013; Sato *et* *al,* 2018). Hence, these circumstances necessitate therapy approaches targeting the malfunctioning immune response. Therefore, inhibiting sustained inflammation represents a plausible therapeutic target to treat a broad range of retinal pathologies (Langmann, 2007).

Here, we comprehensively summarize the role of the three key innate immune pathways in the most common retinal degenerative diseases. Furthermore, we comment on recent developments in preclinical models targeting these pathways and summarize the current status of clinical trials.

## Targeting mononuclear phagocytes in retinal degenerative diseases

### Mononuclear phagocytes in the healthy and diseased retina

Cells of the mononuclear phagocyte (MP) lineage include circulating blood monocytes, tissue‐resident macrophages, dendritic cells, and microglia (Chow *et* *al,* 2011). These cells can be differentiated by their ontogeny, location, function, and phenotype (Guilliams *et* *al,* 2014). MP ontogeny is a research area with much controversy; however, fate‐mapping studies have established that unlike blood‐borne monocyte‐derived macrophages, microglia originate from primitive myeloid progenitors in the extra‐embryonic yolk sac which migrate into the CNS before the blood–brain barrier (BBB) is established (Ginhoux *et* *al,* 2010). Once the tissue is matured, the self‐renewing microglia population is maintained in the brain parenchyma and the retina throughout the entire life span where they compose the resident immune cells (Réu *et* *al,* 2017). Interestingly, in the adult retina, microglia replenish from two distinct extra‐retinal sources as shown by pharmacologic depletion using the selective CSF1R inhibitor PLX5622 (Huang *et* *al,* 2018a). Unlike brain microglia, the cells in the retina were not repopulated from nestin‐positive precursors (Huang *et* *al,* 2018b). Instead, replenished microglia in the retina displayed a dual extra‐retinal origin and long‐distance migration ability. First, the residual microglia in the optic nerve repopulate the retina along the center‐to‐periphery axis, and second, macrophages from the ciliary body and iris relocate to the periphery and migrate toward the center. Furthermore, repopulated microglia fully restore the broad functionalities of naive microglia (Huang *et* *al,* 2018a; Zhang *et* *al,* 2018). These repopulation mechanisms are mainly regulated by the neuronal chemokine CX3CL1 and its receptor (CX3CR1) in microglia (Zhang *et* *al,* 2018).

In the mature retina, microglia reside in the inner and outer plexiform layers and form a sophisticated network of non‐overlapping cells (Hume *et* *al,* 1983). Here, these cells exhibit an abundantly ramified morphology spanning the complete nuclear layers with their long protrusions (Karlstetter *et* *al,* 2015). The dynamic nature of microglia allows them to execute housekeeping functions. The most crucial role is the constant active surveillance of retinal homeostasis where they are indispensable for the immune response and synaptic pruning and transmission (Schafer *et* *al,* 2012; Wang *et* *al,* 2016a).

**Figure 1:**
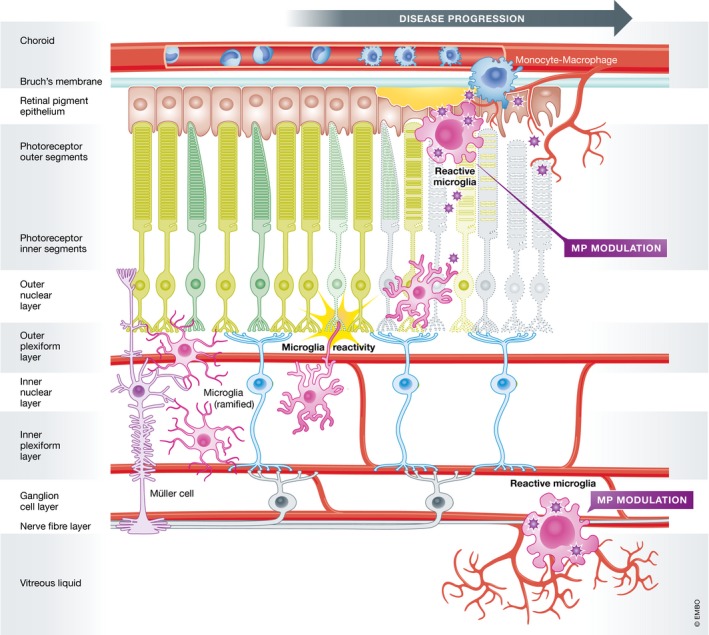
Schematic representation of microglial activity in the retina Under homeostatic conditions, resident microglia mainly populate the plexiform layers. With their long protrusions, they continuously scan their environment and phagocytose cell debris. Different insults leading to abnormal cell functions or degeneration in the RPE, the photoreceptor layer, and the ganglion cell layer rapidly alert microglia. Resident microglia migrate to the lesion sites, where they transform into amoeboid full‐blown phagocytes and recruit macrophages from the periphery. Modified from Karlstetter *et* *al* (2010).

In order to sense the environment for endogenous or exogenous non‐physiological stimuli, microglia engage surface receptors or pattern recognition receptors (PRRs; Kettenmann *et* *al,* 2011; Kigerl *et* *al,* 2014). These surface receptors ligate complement components, cytokines, chemokines, and damage‐ or pathogen‐associated molecular patterns (DAMPs/PAMPs; Karlstetter *et* *al,* 2015). In the event of an insult, microglia sense the danger signals and respond by retracting their surveilling processes concomitant with upregulating the expression of surface receptors (Jurgens & Johnson, 2012). Furthermore, they proliferate and migrate to the site of damage, while releasing pro‐inflammatory cytokines and ROS to neutralize the damage (Ferrer‐Martin *et* *al,* 2015). Moreover, their phagocytic capacity is significantly enhanced to effectively clear debris and prevent accumulation of waste products (Kohno *et* *al,* 2014). In the healthy retina, the insult is rapidly neutralized, the damaged tissue is repaired, and a return to homeostasis is achieved with only very little retinal remodeling (Chen *et* *al,* 2012). This finite microglial activation is beneficial since the toxicity associated with the immune response is outweighed by the toxicity produced due to the noxious insult (Guillonneau *et* *al,* 2017). However, under aggravated conditions owing to genetic predispositions or high glucose levels, microglial activation persists (Gupta *et* *al,* 2003; Omri *et* *al,* 2011). Non‐resolving inflammation causes terminal damage since the increased release of neurotoxic by‐products and the lack of regenerative capacity prevent retinal recovery (Chen & Xu, 2015). Furthermore, over‐reactive microglia are not able to distinguish between stressed and apoptotic cells, and thus phagocyte viable neurons as well (phagoptosis; Brown & Neher, 2012, 2014). Under these circumstances, neurotoxic microglia accumulate at the site of damage and fail to return to their homeostatic state (Tang & Kern, 2011; Ardeljan & Chan, 2013). Additionally, by the secretion of chemokines such as CCL2, microglia attract further phagocytes, which include infiltrating monocytes and choroidal macrophages due to the leakage of the BBB and the newly formed blood vessels (Caicedo *et* *al,* 2005; Sennlaub *et* *al,* 2013). Indeed, such changes in retinal microglia morphology, location, and infiltration of macrophages are common hallmarks of AMD, DR, and hereditary retinopathies (Fig 1; Karlstetter *et* *al,* 2015; Guillonneau *et* *al,* 2017).

Early aspects of DR consist of microaneurysms and intraretinal microvascular anomalies (non‐PDR), while PDR involves neovascularization and preretinal and vitreal hemorrhages (Das *et* *al,* 2015; Lechner *et* *al,* 2017b). Due to these clinical features, DR was only regarded as a vascular complication; however, recent investigations have identified inflammation as an important contributing factor in disease development (Adamis, 2002; Rangasamy *et* *al,* 2012). Elevation in systemic and local chemokine concentration is present in patients suffering from DR (Petrovic *et* *al,* 2010; Koleva‐Georgieva *et* *al,* 2011; Suzuki *et* *al,* 2011). Several authors could demonstrate increased levels of TNF‐α, IL‐8, CCL2, IL‐1β, and IL‐6 in the vitreous fluid (Demircan *et* *al,* 2006; Murugeswari *et* *al,* 2008; Boss *et* *al,* 2017). Furthermore, peripheral monocytes from DR patients secrete high amounts of IL‐1β, IL‐6, TNF‐α, IL‐8, and IL‐1ra (Hatanaka *et* *al,* 2006; Bradshaw *et* *al,* 2009). These inflammatory cytokines are associated with phagocyte reactivity and serve as chemoattractants for invading macrophages. Leakage of the retinal vasculature is mainly induced by VEGF, but also involves TNF‐α, which decreases the expression of tight junction proteins ZO‐1 and claudin‐5 (Behl *et* *al,* 2008; Aveleira *et* *al,* 2010). The increase in endothelial cell permeability then leads to further immune cell recruitment and disease manifestation. Indeed, histopathological analysis of eyes from patients with non‐PDR and PDR exhibited increased numbers of hypertrophic microglia which correlated with disease severity (Zeng *et* *al,* 2008). MPs were clustered around retinal hemorrhages and microaneurysms. In late stages, increased numbers of phagocytes were present in the ganglion cell layer, in the newly formed blood vessels, and around the optic nerve head (Zeng *et* *al,* 2008). Also, there is ample evidence that hyperglycemia induces immune cell reactivity through oxidative stress or indirectly via effects mediated by stressed retinal cells in the proximity (Du *et* *al,* 2002; Rashid *et* *al,* 2018). Oxidative stress in hyperglycemia is driven by accelerated free radical production concomitant with compromised antioxidant generation (Cameron *et* *al,* 1994; Tomlinson & Gardiner, 2008). These circumstances cause translocation of nuclear factor‐kappa B (NF‐κB), pro‐inflammatory cytokine expression, and MP activation (Nishikawa *et* *al,* 2000).

Age‐related macular degeneration, the leading cause of vision loss in the elderly, is a disease of the macula–RPE–choroid interface (Bhutto & Lutty, 2012; Ardeljan & Chan, 2013; Biesemeier *et* *al,* 2014). The RPE is essential for the maintenance and survival of photoreceptor cells by engulfing and degrading shedded photoreceptor disks and protecting the retina against light and oxidative stress (Young, 1967; Boulton, 2013; Mazzoni *et* *al,* 2014). These circumstances require a high metabolic activity rendering the RPE very susceptible to oxidative damage (Mitter *et* *al,* 2012). Oxidative stress is therefore a risk factor for AMD and hence needs rapid neutralization to ensure proper vision (Jarrett & Boulton, 2012). Indeed, the RPE engages in autophagy and mitophagy, directing ROS‐producing mitochondria to lysosomes, as defense mechanisms against ROS increase (Lee *et* *al,* 2012; Mitter *et* *al,* 2012; Ferguson & Green, 2014). Mitter *et* *al* (2014) found an age‐related increase in autophagosomes and proteins involved in autophagy in the RPE; however, these factors were significantly reduced in human AMD donor eyes. Deteriorated autophagy concomitant with increased accumulating ROS results in aggregation of damaged organelles and toxic by‐products including the photoreactive age‐pigment lipofuscin (Wang *et* *al,* 2009; Mitter *et* *al,* 2014). These deposits become apparent as drusen located in the sub‐RPE area and subretinal space during early AMD (Ishibashi *et* *al,* 1986; Abdelsalam *et* *al,* 1999; Gupta *et* *al,* 2003; Fritsche *et* *al,* 2016). Strikingly, drusen components include lipoproteins and complement factors serving as attractants and activators of MPs (Penfold *et* *al,* 1985; Johnson *et* *al,* 2000; Hageman *et* *al,* 2001; Nozaki *et* *al,* 2006; Buschini *et* *al,* 2011). Indeed, bloated phagocytic microglia were found to closely associate with drusen in AMD patients (Gupta *et* *al,* 2003). While the elimination of retinal debris by MPs is principally positive, impaired function during aging of these cells leads to sustained pro‐inflammatory environment (Streit *et* *al,* 2004; Chan‐Ling *et* *al,* 2007; Damani *et* *al,* 2011). A comparative transcriptome analysis of AMD and normal human donor eyes demonstrated a significant over‐expression of immune‐related transcripts including complement and chemokine mRNAs in all AMD samples (Newman *et* *al,* 2012). Furthermore, high levels of CCL2 and VEGF, two cytokines involved in MP recruitment and choroidal neovascularization (CNV), are present in ocular fluids from neovascular AMD patients (Fauser *et* *al,* 2015; Lechner *et* *al,* 2017a). As a consequence, accumulating subretinal microglia can directly induce death of nearby photoreceptors. This suggests that microglial reactivity is a driving force in photoreceptor demise and disease manifestation.

Unlike AMD, which is a multifactorial disease, hereditary degenerations of the human retina are mostly monogenic. The majority of the documented mutations are associated with genes expressed in photoreceptors and RPE (Karlstetter *et* *al,* 2015; RetNet, 2018). RP, the most common form of hereditary retinal degeneration, is characterized by night blindness and tunnel vision due to rod demise (Hartong *et* *al,* 2006). Even more, late stages of RP are characterized by central vision loss attributed to the secondary death of cone photoreceptors (Hartong *et* *al,* 2006). Involvement of MPs was proven by analyzing retinal sections with concentric RP demonstrating bloated microglia in the photoreceptor layer with rhodopsin‐positive inclusions (Gupta *et* *al,* 2003; Zhao *et* *al,* 2015a,b,c). The authors hypothesized that activated phagocytes release pro‐inflammatory cytotoxic factors that subsequently trigger the death of adjacent cones. The resulting pro‐inflammatory environment leads to further recruitment of MPs which, when overly activated, are unable to discriminate between dead versus stressed‐but‐viable neurons and hence engage in phagoptosis (Brown & Neher, 2012, 2014). Indeed, co‐staining of rhodopsin with apoptosis markers in mouse models of RP demonstrated rhodopsin‐positive inclusions that were mostly TUNEL‐negative (Zhao *et* *al,* 2015a,b,c). Furthermore, microglial phagocytosis in the ONL corresponded to concurrent exposure of phosphatidylserine which serves as an “eat‐me” signal in stressed rods. Also, microglia were found to upregulate lactadherin/milk fat globule‐EGF factor 8 protein (MFG‐E8), a “bridging” molecule between phagocytes and phosphatidylserine on neurons to facilitate rapid engulfment and internalization of stressed neurons (Neniskyte & Brown, 2013).

### Therapeutic strategies targeting mononuclear phagocytes in preclinical models of retinal degenerative diseases

As discussed above, MP activation in the retina is initiated either through direct recognition of immune triggers, such as DAMPs/PAMPs, chemoattractants, and complement components, or indirectly by sensing an amplitude of stressors in the surrounding environment including ROS and “eat‐me” signals from dying cells. Overshooting MP reactivity often leads to tissue damage, but their depletion does not always result in tissue homeostasis (Zhao *et* *al,* 2015a,b,c). Several studies point out that microglia are indispensable for the maintenance of synaptic structures in the adult CNS. Their depletion in the mature CNS can cause deficits in learning tasks and a significant reduction in motor‐learning‐dependent synapse formation (Parkhurst *et* *al,* 2013). More importantly, phagocyte ablation in the adult retina leads to the degeneration of photoreceptor synapses in the outer plexiform layer and hence a functional deterioration in retinal light responses (Wang *et* *al,* 2016a). Therefore, effective immunomodulatory compounds should dampen the overt pro‐inflammatory response of retinal phagocytes but preserve their homeostatic functions which are vital for retinal integrity.

Mononuclear phagocytes are composed of a heterogeneous population with diverse functionalities (Hanisch, 2013). What remains inconclusive is whether the beneficial and detrimental effects carried out by reactive MPs are executed by the same population or by distinct subtypes. Distinguishing these cell populations is a challenging task. For instance, infiltrating macrophages cannot be easily separated from resident microglia in laser‐induced CNV in mice. Laser‐induced CNV is extensively applied in retinal research since it recapitulates several main features of exudative AMD (Lambert *et* *al,* 2013). The laser impact results in the rupture of Bruch's membrane, a rapid recruitment of MPs, and penetration of choroidal capillaries into the avascular retina within a few days. In contrast, alternative models for experimental CNV that involve injections of pro‐angiogenic substances have a much lower incidence of neovascularization (Shah *et* *al,* 2015). In order to specifically target resident microglia and to distinguish them from short‐lived infiltrating cells in this model, we used tamoxifen‐inducible conditional Cx3cr1^CreER^ mice to delete the floxed gene for interferon‐α/β receptor 1 (Ifnar1; Luckoff *et* *al,* 2016). These Cx3cr1^CreER^:Ifnar1^fl/fl^ mice were subjected to laser injury 4 weeks after tamoxifen injection when monocyte‐derived macrophages were already washed out and replaced. However, no differentiation between retinal microglia and potentially long‐lived tissue‐resident macrophages in the periphery could be achieved (Reyes *et* *al,* 2017). Another elegant approach for cell discrimination is to use fate‐mapping combined with endogenous genetic reporters and multiple expression markers. O'Koren *et* *al* (2016) demonstrated that retinal microglia have a unique CD45(low) CD11c(low) F4/80(low) I‐A/I‐E(−) signature which is conserved in the steady state and during retinal injury. By investigating these cells, the authors found that microglia migrate to the photoreceptor outer segments while monocyte‐derived macrophages appear throughout the entire retina (Reyes *et* *al,* 2017). For further insights into microglia heterogeneity, the reader is directed to other excellent reviews (Hanisch, 2013; Reyes *et* *al,* 2017).

Genetic mouse models combined with experimental approaches mimicking retinal degenerative diseases have greatly expanded our knowledge on the mechanisms involved in retinal MP activation (Luckoff *et* *al,* 2017). In the following section, we present main concepts for microglia‐related immunomodulation. These strategies involve the prevention and/or resolution of retinal degeneration and neovascularization by (i) targeting activating and inhibitory cell surface receptors, (ii) modulating intracellular molecules, and (iii) controlling released inflammatory mediators.

#### Purinergic receptors

**Figure 2:**
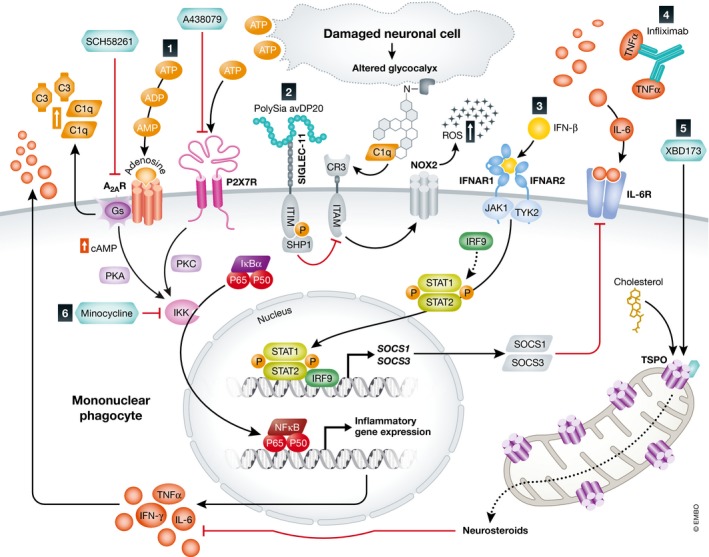
Activation pathways and therapy targets for mononuclear phagocytes (1) The purines ATP and adenosine ligate to their receptors P2X7 and A2AR, respectively, which act through PKA/PKC signaling and thereby activate IKK. IKK aids to cleave and translocate NF‐κB into the nucleus and induce inflammatory gene expression and cytokine release. A2AR additionally potentiates the expression of the complement component C3 and the opsonin C1q. Neuroprotective effects were observed when A2AR and P2X7R were blocked using the selective inhibitors SCH58261 and A438079, respectively. (2) Intravitreal administration of polySia avDP20 compensates for desialylated neurons by binding to its receptor SIGLEC‐11 and inhibiting neurotoxic inflammation through its ITIM domain. During inflammation, desialylated neurons activate CR3 associated ITAM and thereby trigger excessive release of ROS via NOX2. (3) The IFN‐β pathway involves STAT1‐ and STAT2‐induced SOCS1/SOCS3 release which reduces cytokine expression through a negative feedback and inhibits IL‐6 signaling. (4) Neutralizing antibodies such as infliximab aid to neutralize cytosolic TNF‐α. (5) TSPO ligands such as XBD173 stimulate the production of neurosteroids which limit the release of pro‐inflammatory cytokines. (6) Minocycline is a potent inhibitor of NF‐κB signaling.

The purines adenosine triphosphate (ATP) and adenosine serve as neuro‐ and gliotransmitters in the retina contributing to the bidirectional neuron–glia communication as well as the cross‐talk between photoreceptors and the RPE (Newman, 2006; Housley *et* *al,* 2009; Wurm *et* *al,* 2010). Physiologically, purines are tonically released in the dark; however, this release is elevated when neurons are active (Khakh & North, 2006; Uckermann *et* *al,* 2006; Niyadurupola *et* *al,* 2013). Growing evidence suggests that dysregulated purinergic signaling contributes to gliosis in the diseased retina (Sanderson *et* *al,* 2014). Degenerating cells or elevated glucose levels increase the extracellular ATP concentration, which activates the P2X7 receptor (P2X7R) on MPs and induces a chemokine release through PKC/MAP kinase pathway activation (Fig 2; Potucek *et* *al,* 2006; Costa *et* *al,* 2009; Shiratori *et* *al,* 2010; He *et* *al,* 2017). ATP stimulation evokes the release of pro‐inflammatory cytokines IL‐6, TNF‐α, and CCL2 in primary microglia, which was absent when P2X7 was deleted (Morigiwa *et* *al,* 2000; Shieh *et* *al,* 2014). In a murine model of axonal injury that culminates in the death of retinal neurons, P2X7‐deficient mice exhibited a delayed loss of retinal neurons and a decrease in phagocytic microglia (Nadal‐Nicolas *et* *al,* 2016). Moreover, intravitreal administration of the selective P2X7 antagonist A438079 delayed axotomy‐induced ganglion cell death.

Adenosine is a neuromodulator critically involved in neurodegenerative diseases (Yu *et* *al,* 2004; Kalda *et* *al,* 2006). It modulates microglial reactivity mainly through the activation of its G‐protein‐coupled receptor A2A (A2AR; Fig 2; Cunha, 2001; Canas *et* *al,* 2009). Strikingly, agonists of A2AR potentiate LPS‐induced microglial reactivity (Saura *et* *al,* 2005). Conversely, its pharmacological inhibition results in neuroprotective effects by attenuating microglial NO production and modulating cyclooxygenase‐2 (COX2) expression in a rat model of striatal excitotoxicity (Saura *et* *al,* 2005; Minghetti *et* *al,* 2007). Similarly, inhibition of A2AR prevents microglial reactivity in mice that were subjected to intraperitoneal LPS injection (Rebola *et* *al,* 2011) and selective A2AR blockade reduces NO production in microglia (Madeira *et* *al,* 2015). In a retinal degeneration model of transient ischemia–reperfusion, intravitreal injection of the A2AR‐blocking compound SCH58261 attenuated neuronal loss by inhibiting microglial reactivity (Madeira *et* *al,* 2016). In microglia, A2AR clearly facilitates the release of cytokines and NO production, which are likely induced through increased cAMP levels and the activation of protein kinase A (PKA; Fig 2; Moreau & Huber, 1999). Subsequent activation of MAP kinases including extracellular signal‐regulated kinase (ERK) 1/2 and IkappaB kinase (IKK) then induces altered gene expression (Kyriakis & Avruch, 2001; Schulte & Fredholm, 2003; Chio *et* *al,* 2004; Dang *et* *al,* 2014). Recently, we showed that A2AR antagonism also limits complement and inflammasome activation (Madeira *et* *al,* 2018). The exposure of human microglia to RPE cell debris induced activation of the complement cascade which is strongly associated with the pathogenesis of AMD (Zipfel & Skerka, 2009; Schick *et* *al,* 2017). Inhibition of A2AR prevented this change in microglial complement activation as well as inflammasome activation in ARPE‐19 cells that were exposed to conditioned media from activated microglia treated with the A2AR blocker. Therefore, selective A2AR antagonists could serve as tools to modulate microglial activity and limit RPE inflammatory response.

#### CD200 receptor

CD200 is a transmembrane glycoprotein expressed on the vascular endothelium, photoreceptors, RPE, and ganglion cells. Its respective receptor is expressed on microglia which, upon ligand binding, provides a potent quiescence signal (Dick *et* *al,* 2001, 2003). Deficiency in CD200 signaling is associated with microglial proliferation and strong iNOS expression, indicating a latent pro‐inflammatory condition (Dick *et* *al,* 2003). In an animal model of uveoretinitis, CD200 deficiency caused increased numbers of microglia concomitant with increased expression of iNOS (Broderick *et* *al,* 2002). Similarly, in the murine laser model of exudative AMD, CD200R knockout animals displayed significantly increased new vessel formation and elevated levels of VEGF‐A, arginase 1, and IL‐1β (Horie *et* *al,* 2013). Conversely, intravitreal injection of the CD200R agonistic monoclonal antibody DX109 diminished microglial reactivity and macrophage infiltration, thereby suppressing pathological angiogenesis and vascular damage (Horie *et* *al,* 2013). Also, systemic administration of DX109 was effective in suppressing IFN‐γ‐mediated phagocyte activation and protected against tissue damage during experimental autoimmune uveoretinitis (Copland *et* *al,* 2007). Hence, CD200R agonists could be used to diminish pro‐angiogenic and pro‐inflammatory gene expression which prevents pathological angiogenesis.

#### Polysialic acid receptors

In the vertebrate CNS, polysialic acid (polySia) caps consistent of α‐8‐linked N‐acetylneuraminic acids are commonly attached to glycoproteins of healthy neurons (Schnaar *et* *al,* 2014; Hildebrandt & Dityatev, 2015). PolySia can alleviate neurotoxicity via binding to sialic acid‐binding immunoglobulin‐like lectin 11 (SIGLEC‐11), a primate lineage‐specific receptor (Angata *et* *al,* 2002; Linnartz‐Gerlach *et* *al,* 2014). Interestingly, some strains of neuroinvasive bacteria can also produce capsular polysaccharide identical to polySia in order to escape immune recognition in the human host (Troy, 1992; Schwarz *et* *al,* 2017). Ectopic expression of human SIGLEC‐11 in cultured murine microglia suppressed the LPS‐induced transcription of the pro‐inflammatory mediators IL‐1β and iNOS (Wang & Neumann, 2010). SIGLEC‐11 mediates immune inhibition through its cytosolic immunoreceptor tyrosine‐based inhibition motif (ITIM; Fig 2). Binding of polySia to its receptor leads to phosphorylation of ITIM (Linnartz & Neumann, 2013). Successive recruitment of the second messenger SHP1 dephosphorylates the intracellular immunoreceptor tyrosine‐based activation motif (ITAM), thus limiting a number of downstream pathways linked to the phagocytosis of neurites and NADPH oxidase (NOX2)‐mediated production of ROS (Fig 2; Graham *et* *al,* 2007; Hamerman *et* *al,* 2009). Under pathological conditions, immune cells secrete neuraminidases which cleave sialic acid residues on neurons (Amith *et* *al,* 2010; Pshezhetsky & Hinek, 2011; Nomura *et* *al,* 2017). Desialylated neurons are consequently opsonized by complement component C1q, which is produced and secreted by microglia (Linnartz *et* *al,* 2012; Madeira *et* *al,* 2018). Indeed, soluble sialic acid residues accumulate in serum, and C1q is found in the retina during early stages of AMD (van der Schaft *et* *al,* 1993; Goswami *et* *al,* 2003). Subsequently, the opsonized glycocalyx is recognized by complement receptor 3 (CR3) coupled to ITAM leading to phagocytosis of the neuronal structures (Fig 2; Linnartz *et* *al,* 2012). Intriguingly, blockage of CR3 prevented neurite phagocytosis by microglia, which was as seen when polySia was removed from cultured neurons by treatment with sialidases (Wang & Neumann, 2010).

These results point toward a polySia‐based therapy to target inflammation. Consistently, studies showed that nanomolar concentrations of low molecular weight polySia with average degree of polymerization of 20 (polySia avDP20) significantly reduced pro‐inflammatory gene transcription, abnormal phagocytosis, and oxidative burst in human macrophages challenged with LPS or amyloid‐β1–42 (Shahraz *et* *al,* 2015). Furthermore, we used humanized transgenic mice expressing human SIGLEC‐11, subjected them to laser injury, and treated them with intravitreal injections of polySia avDP20 (Karlstetter *et* *al,* 2017). Already low doses of polySia avDP20 significantly reduced microglial activation and vascular leakage by reducing TNF‐α and VEGF‐A levels as well as superoxide production (Fig 2). As a second mechanism of action, independent from SIGLEC‐11 signaling, higher doses of polySia avDP20 blocked alternative complement activation and reduced membrane attack formation in the diseased retina (Karlstetter *et* *al,* 2017).

#### Interferon‐β (IFN‐β)

Important evidence for potent immunomodulatory effects of IFN‐β on brain microglia came from gene deletion studies in experimental autoimmune encephalomyelitis (EAE) mouse models. Animals lacking either the Ifn‐β gene or its cognate interferon‐α/β receptor (Ifnar) exhibited elevated microglial reactivity concomitant with an even severe EAE disease phenotype when compared to wild‐type controls (Teige *et* *al,* 2003; Prinz *et* *al,* 2008). More importantly, IFN‐β treatment in a multiple sclerosis patient completely reversed subfoveal neovascularization and choroiditis emphasizing the therapeutic potential of IFN‐β for inflammatory and vascular diseases of the eye (Cirino *et* *al,* 2006).

We have therefore studied whether IFN‐β therapy could have beneficial immunomodulatory effects in the laser CNV model for exudative AMD. We demonstrated that systemic administration of IFN‐β not only inhibited MP reactivity and macrophage recruitment but also reduced vascular leakage and neoangiogenesis (Luckoff *et* *al,* 2016). The immune cell reactivity was evaluated by counting the total number of reactive MPs in the laser spot and their ramification status. Both parameters were significantly affected by IFN‐β treatment. Conversely, genetic deletion of Ifnar1 in mice resulted in aggravated disease after laser treatment. Similar results were obtained with a microglia‐specific conditional deletion of IFN‐β signaling (Cx3cr1^CreER^:Ifnar1^fl/fl^). Our results provide a mechanistic explanation for earlier publications where damage associated with laser photocoagulation in rabbits and monkeys was successfully treated with IFN‐β (Tobe *et* *al,* 1995; Kimoto *et* *al,* 2002). Apart from affecting microglial reactivity by blocking the production of neurotoxic superoxide radicals, IFN‐β also promotes RPE homeostasis and suppresses proliferative activity of endothelial cells (Kimoto *et* *al,* 2002; Jin *et* *al,* 2007).

Despite the clear indications of a protective IFN‐β signaling in retinal microglia, the exact molecular pathways remain poorly understood. IFN‐β signaling involves the transcription of suppressor of cytokine signaling 1 (SOCS1) and SOCS3 by translocating the transcription factors STAT1 and STAT2 into the nucleus (Rashid *et* *al,* 2018; Fig 2). SOCS1 and SOCS3 expression is known to engage in inhibitory signals to mitigate microglial activation and prevent cell toxicity (Kimura *et* *al,* 2005; Baker *et* *al,* 2009; McCormick & Heller, 2015). Supporting evidence comes from a study where SOCS3 deficiency in myeloid cells exaggerated retinal degeneration and accelerated retinal angiogenesis in a murine model of uveoretinitis (Chen *et* *al,* 2018). In these mice, SOCS3‐deficient retinas demonstrated higher levels of pro‐inflammatory cytokines IL‐1β, TNF‐α, and IFN‐γ as well as angiogenic factors including VEGF‐A. Similarly, SOCS1 protected retinal cells from staurosporine‐ and H_2_O_2_‐induced apoptosis (Yu *et* *al,* 2011). Also, members of the SOCS family are potentially key physiological negative regulators of IL‐6 signaling in macrophages (Croker *et* *al,* 2003; Wilson, 2014; Fig 2).

#### TSPO ligands

Translocator protein 18 kDa (TSPO) is a highly conserved 5α‐helical transmembrane protein located on the outer mitochondrial membrane (Girard *et* *al,* 2012). Highly induced TSPO protein expression is predominantly found in activated microglia during various neuropathological conditions (Daugherty *et* *al,* 2013; Karlstetter *et* *al,* 2014; Rashid *et* *al,* 2018). Concomitantly, astrocytes and Müller cells upregulate the secretion of an endogenous TSPO ligand, diazepam binding inhibitor (DBI) protein which is sensed by microglia and serves to limit the magnitude of microglial reactivity by inducing feedback regulation (Wang *et* *al,* 2014). Finally, triakontatetraneuropeptide (TTN), the biologically active cleavage product of DBI, triggers the transformation of activated microglia to baseline quiescence (Wang *et* *al,* 2014).

Based on this concept of feedback regulation, synthetic TSPO ligands were effective immunoregulators in various animal models for neurological diseases including Alzheimer's disease, multiple sclerosis, and anxiety disorders (Rupprecht *et* *al,* 2009; Barron *et* *al,* 2013). In our study on retinal degeneration, we tested the ability of the specific TSPO ligand XBD173 to dampen microglial reactivity in the acutely white light‐damaged mouse retina (Fig 2). In this model, exposure to intense white light leads to a significant loss of photoreceptor cells and thinning of the outer nuclear layer within a few days after dark adaptation and light exposure (Wenzel *et* *al,* 2005). We found that systemic administration of XBD173 markedly limited the accumulation of amoeboid microglia in the outer retina and protected from overt cell death (Scholz *et* *al,* 2015a). Mechanistically, XBD173 efficiently suppressed pro‐inflammatory gene expression in cultured microglia and reduced neuronal cell death in microglia‐conditioned medium (Karlstetter *et* *al,* 2014). Moreover, XBD173 triggered a neuroprotective microglia phenotype in explanted organotypic mouse retinal cultures (Karlstetter *et* *al,* 2014). These effects mediated by XBD173 were prevented upon blocking the enzymatic conversion of cholesterol to pregnenolone (Fig 2), which can be converted to progesterone, a potent neurosteroid with pleiotropic neuroprotective properties (Pettus *et* *al,* 2005; Guennoun *et* *al,* 2015; Cai *et* *al,* 2018). In *rd*1 mice, a model for retinitis pigmentosa, oral progesterone treatment decreased gliosis and cell death leading to improved retinal function (Sanchez‐Vallejo *et* *al,* 2015). Similarly, TTN stimulation of microglia increased levels of dehydroepiandrosterone, an effective anti‐inflammatory neurosteroid (Wang *et* *al,* 2014), and the TSPO ligand Ro5‐4864 effectively reduced diabetic neuropathy through a local increase in neurosteroids (Giatti *et* *al,* 2009). These findings clearly support the concept that TSPO exerts its neuroprotective effects by modulating neuronal steroidogenesis.

#### Minocycline

Minocycline is a membrane‐permeable semi‐synthetic tetracycline derivative with strong neuroprotective and immunomodulatory effects (Garrido‐Mesa *et* *al,* 2013). Minocycline blocks microglial activation in response to a variety of inflammatory stimuli by inhibiting Toll‐like receptor 2 (TLR2) and TLR4 signaling (Nikodemova *et* *al,* 2006; Halder *et* *al,* 2013). TLRs induce a potent immune response upon recognition of PAMPs (Uematsu & Akira, 2006). Receptor signaling triggers cytokine production through translocation of NF‐κB into the nucleus which is essential for the defense of the host cell (Beutler, 2004). Increased expression of TLR2 and TLR4 concomitant with elevated NF‐κB levels is often found in human monocytes under conditions of hyperglycemia (Mohammad *et* *al,* 2006; Dasu *et* *al,* 2008). In the mouse retina, pre‐diabetic conditions and high‐fat diet caused TLR4‐dependent activation of microglia ad macrophages concomitant with vision loss (Lee *et* *al,* 2015). Microglia themselves can experience necroptosis, a form of inflammatory cell death, through TLR4 activation in *rd*1 mice, thereby exacerbating retinal inflammation and damage (Huang *et* *al,* 2017).

Minocycline potently inhibits NF‐κB transcriptional activity by blocking the degradation of IκBα (Nikodemova *et* *al,* 2006; Fig 2). Systemic minocycline therapy in light‐damaged mice reduced pro‐inflammatory cytokine release, prevented microgliosis, and preserved photoreceptor function in the retina (Zhang *et* *al,* 2004; Scholz *et* *al,* 2015b). Similarly, minocycline inhibited microglial reactivity and photoreceptor apoptosis in the *rd*10 mouse model of human RP (Peng *et* *al,* 2014). In a streptozotocin (STZ)‐induced rat model of DR, minocycline blocked microglial COX2 expression and prevented the release of IL‐1β and TNF‐α with concomitant reduction in caspase‐3‐mediated apoptosis (Krady *et* *al,* 2005). Recent evidence also suggests that minocycline can block the expression of PARP1, a chromatin‐associated enzyme which promotes the expression of IL‐1β and TNF‐α in glial cells, and thereby reduces retinal apoptosis (Wu *et* *al,* 2015).

#### Cytokine inhibition

Enhanced levels of pro‐inflammatory cytokines are involved in AMD (de Oliveira Dias *et* *al,* 2011), RP (He *et* *al,* 2015), and DR (Patel *et* *al,* 2008). Specifically, the cytokines TNF‐α, IL‐1β, and IL‐6 are significantly elevated in retinal pathologies at the time point of immune cell reactivity (Armstrong *et* *al,* 1998; Oh *et* *al,* 1999; Seddon *et* *al,* 2005; Poon *et* *al,* 2015; Zhao *et* *al,* 2015a). Therefore, scavenging of cytokines has been a valid therapeutic concept in these retinal pathologies.

Several TNF‐α‐inhibiting antibodies have been developed and tested as potential therapy options for retinal degenerations including preclinical models for AMD, glaucoma, and ischemic retinopathy (Al‐Gayyar & Elsherbiny, 2013). Secretion of TNF‐α by phagocytes stimulates VEGF production in RPE and promotes angiogenesis, hence being a candidate target for treating AMD and DR (Cousins *et* *al,* 2004; Regatieri *et* *al,* 2009). TNF‐α is also a negative regulator of the RPE transcription factor orthodenticle homeobox 2 (OTX2) which orchestrates expression of critical genes involved in proper retinal function (Mathis *et* *al,* 2017). In various rodent models, researchers demonstrated positive effects of intravitreal injections of the TNF‐α antibodies (Shi *et* *al,* 2006; Regatieri *et* *al,* 2009). Histopathological findings confirmed that CNV lesions in treated mice were smaller in size compared to the control animals (Shi *et* *al,* 2006). Furthermore, intravitreal injection of low doses of infliximab (10–40 μg) abates the cytokine availability and modulates angiogenesis (Fig 2; Regatieri *et* *al,* 2009). Studies in monkey demonstrated that intravitreal injections of adalimumab and the single‐chain antibody fragment ESBA105, both potent TNF‐α inhibitors, resulted in CNV reduction, whereas topical treatment had only weak effects (Lichtlen *et* *al,* 2010).

IL‐1β is strongly involved in neovascularization by triggering the release of angiogenic factors (Joyal *et* *al,* 2011; Horie *et* *al,* 2013; Rivera *et* *al,* 2013). Thus, IL‐1β induces a robust release of semaphoring‐3A in retinal ganglion cells and RPE in an oxygen‐induced retinopathy model (Martin *et* *al,* 2004; Joyal *et* *al,* 2011). Sema3A critically contributes to vascular decay and misguided revascularization (Rivera *et* *al,* 2013). Furthermore, excessive IL‐1β release induces P2X7R expression on monocytes, thereby triggering further IL‐1β release and retinal apoptosis (Giuliani *et* *al,* 2017). Inhibition of IL‐1β or P2X7R completely prevented the inflammation‐associated photoreceptor demise (Hu *et* *al,* 2015). Recently, Natoli *et* *al* (2017) showed that inhibition of retinal IL‐1β reduced phagocyte accumulation and photoreceptor death via downregulating chemokine expression by Müller cells and RPE in rats with focal photo‐oxidative damage.

Likewise, high levels of IL‐6 are significantly related to AMD progression and increased in mice with experimentally induced CNV (Seddon *et* *al,* 2005; Izumi‐Nagai *et* *al,* 2007). Systemic administration of the anti‐IL‐6R monoclonal antibody MR16‐1 effectively suppressed the expression of CCL2 and VEGF and reduced macrophage infiltration as well as the CNV area (Izumi‐Nagai *et* *al,* 2007). IL‐6 is known to repress Fas ligand expression in the RPE, which then leads to impaired clearance and accumulation of MPs in the subretinal space (Levy *et* *al,* 2015).

#### Chemokine modulation

Fractalkine or CX3CL1 is a neuronal chemokine which binds to its receptor CX3CR1 on microglia and macrophages (Geissmann *et* *al,* 2003; Wolf *et* *al,* 2013). The tightly regulated cross‐talk between neurons and microglia involving CX3CL1–CX3CR1 has an important role in immunoregulation and neuroprotection in the brain and the retina (Wolf *et* *al,* 2013; Zieger *et* *al,* 2014). Thus, CX3CR1‐deficient mice show a higher susceptibility to subthreshold light challenge leading to the accumulation of subretinal microglia, which can be prevented by keeping the animals in the dark (Combadière *et* *al,* 2007; Chinnery *et* *al,* 2012). Inhibition of CC‐motif chemokine ligand 2 (CCL2) or IL‐1β also prevented inflammatory macrophage recruitment and photoreceptor degeneration in these animals (Sennlaub *et* *al,* 2013; Eandi *et* *al,* 2016). Conversely, positive modulation of CX3CL1–CX3CR1 signaling in the diabetic mouse retina by intravitreal administration of recombinant fractalkine effectively reduced microglial proliferation (Mendiola *et* *al,* 2017).

The otherwise low expression of CCL2 in the retina is strongly enhanced under stressful conditions (Nakazawa *et* *al,* 2007; Chen *et* *al,* 2012). It is primarily secreted by activated microglia to recruit inflammatory monocytes expressing CCR2 (Mizutani *et* *al,* 2012; Sennlaub *et* *al,* 2013). The pro‐inflammatory CCL2/CCR2 axis represents a valid target for inhibition to restore immune balance. Thus, rats receiving intravitreal injection of CCL2 siRNA showed a markedly decreased phagocyte accumulation and photoreceptor apoptosis after light damage (Rutar *et* *al,* 2012). Similarly, CCR2 knockout mice had much lower photoreceptor demise after chronic blue light exposure (Hu *et* *al,* 2016), less pro‐inflammatory cells and CNV in the laser‐damage model (Robbie *et* *al,* 2016), and fewer subretinal macrophages when immunized with carboxyethylpyrrole‐modified albumin as trigger for oxidative stress (Cruz‐Guilloty *et* *al,* 2013).

The secretion of CCL3 by microglia is an early event in the pathologies of the Abca4^−/−^ Rdh8^−/−^ mouse model of Stargardt disease and the Mertk^−/−^ mouse model of RP (Kohno *et* *al,* 2014). Consequently, knockout of CCL3 in these mice resulted in a milder disease form with increased retinal thickness, fewer numbers of subretinal phagocytes, and marked reduction in vascular leakage (Kohno *et* *al,* 2014).

### Mononuclear phagocyte‐targeted therapy in patients with retinal pathologies

#### Hyper‐reflective foci

When monitoring the retina of wet AMD patients using spectral domain optical coherence tomography (SD‐OCT), small, dense particles—thereby referred to as hyper‐reflective foci (HF)—were identified (Framme *et* *al,* 2010; Altay *et* *al,* 2016). Similarly, HF were found in eyes of patients with different stages of diabetic retinopathy (De Benedetto *et* *al,* 2015; Korot *et* *al,* 2016), and a positive correlation between HF number, hard exudate size, disease severity, and inflammation has been recognized (Bolz *et* *al,* 2009; Lammer *et* *al,* 2014; Niu *et* *al,* 2017). Interestingly, the appearance and resolution of HF can be used to complement current diagnostic tools and predict disease progression and therapeutic success (Gallagher *et* *al,* 2007; Coscas *et* *al,* 2013; Abri Aghdam *et* *al,* 2015). These findings led to the assumption that HF may represent either migrating RPE cells or reactive and bloated immune cells (Framme *et* *al,* 2010; Christenbury *et* *al,* 2013; Coscas *et* *al,* 2013; Gocho *et* *al,* 2013). Pang *et al* compared HF with histological analyses in two donor eyes and found cholesterol crystals, indicating that HF are either RPE cells or lipid‐filled phagocytes (Ogino *et* *al,* 2012; Pang *et* *al,* 2015). Thus, the success of phagocyte‐targeting therapies could be potentially assessed by monitoring the presence and number of retinal HF using non‐invasive OCT imaging.

#### Clinical trials

Despite the very promising results in a wide range of preclinical studies targeting retinal phagocytes, only limited data are available from clinical trials. In a phase I/II study, Nussenblatt *et al* compared three immunosuppressive agents—daclizumab, rapamycin, and infliximab—in combination with anti‐VEGF therapy in AMD patients (Nussenblatt *et* *al,* 2010). The authors found that treatment with the anti‐IL‐2 receptor antibody daclizumab, as well as the mTOR inhibitor sirolimus, but not infliximab, decreased the number of anti‐VEGF intravitreal injections. However, no significant changes in visual acuity were evident. In contrast, other studies reported positive effects on vision gain following intravitreal or systemic infliximab treatment in patients with AMD and DR (Sfikakis *et* *al,* 2005, 2010; Theodossiadis *et* *al,* 2009) although some retinotoxicity was found in a small group of patients (Giganti *et* *al,* 2010).

Oral minocycline treatment was tested in five patients with DME and one patient with RP (Cukras *et* *al,* 2012; Baumgartner & Baumgartner, 2013). A 6‐month treatment with minocycline improved visual function, central macular edema, and vascular leakage in the DME patients (Cukras *et* *al,* 2012). The RP patient received minocycline together with the anti‐apoptotic drug deprenyl for 120 months and showed a slower decline in visual field as estimated from the previously documented disease course (Baumgartner & Baumgartner, 2013).

## Targeting the complement system in retinal degenerative diseases

### The complement pathways

The complement system, with over 30 small proteins, is a crucial component of innate immunity. Its origins can be traced back to a billion years ago when primitive proteins evolved to protect cells from pathogens. However, it was only discovered around 125 years ago as a liver‐derived heat‐labile substance circulating in the blood that “complements” antibodies in lytic killing of bacteria, fungi, and viruses.

**Figure 3:**
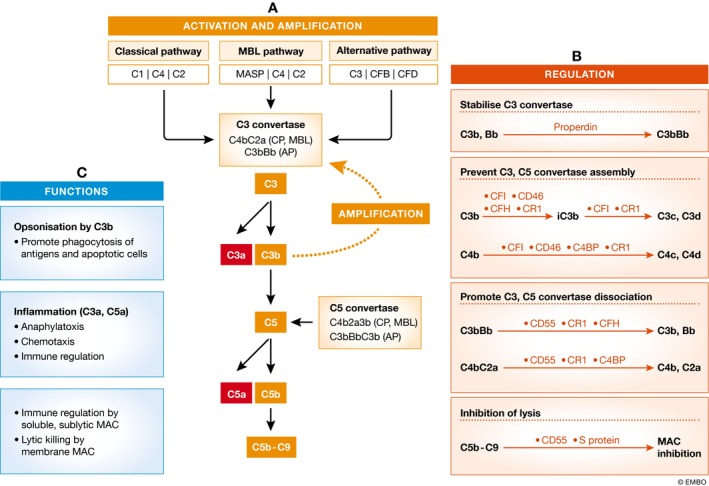
Complement activation, regulation, and immune functions (A) The complement system can be activated by three pathways: the classical pathway (CP), mannose‐binding lectin (MBL) pathway, and the alternative pathway (AP); all lead to the cleavage of C3 and C5 and the formation of C5b‐C9. In the CP and MBL pathways, the C3 and C5 convertases are C4b2a and C4b2a3b, whereas in the AP, they are C3bBb and C3bBbC3b, respectively. Once C3 is cleaved into C3a and C3b, the C3b fragment can form C3bBb to amplify the complement activation cascade. Therefore, even if the initial activation is mediated by CP or MBL pathway, the cascade is ultimately amplified by the AP. (B) The complement activation cascades are regulated at multiple levels. Properdin is the only factor that stabilizes C3bBb and enhances complement activation. CFI, CD46, CFH, CR1, and C4BP prevent the assembly of C3 and C5 convertases by further breaking down C3b and C4b, whereas CD55, CR1, CFH, and C4BP can dissociate C3 and C5 convertases. CD59 and S protein can prevent the assembly of C5b‐C9. (C) Activation of the complement system generates C3a, C3b, and C5a fragments that are actively involved in immune responses. The soluble form of and sublytic levels of C5b‐C9 can regulate immune cell functions, whereas the membrane MACs directly kill pathogens and cells.

The system becomes activated in a cascade fashion when triggered through one or more of the three major pathways: the classical pathway (CP), the mannose‐binding lectin (MBL) pathway, and the alternative pathway (AP; Fig 3). There are two critical steps for the full activation of the complement pathways: C3 cleavage and C5 cleavage by relevant convertases (Fig 3).

These convertases generate (i) C3b that may participate in opsonizing pathogens or dead cells and promoting their clearance, or form C5 convertase in the AP's feedback loop (Fig 3), (ii) C3a and C5a that can induce vasodilation, increase the permeability of small blood vessels, and induce contraction of smooth muscles (Fig 3), and (iii) C5b‐C9, also called membrane attack complex (MAC) that can modulate the immune response or affect cell cycle when released as a soluble form and at sublytic levels (Lueck *et* *al,* 2011; Lakkaraju *et* *al,* 2014) or form transmembrane channels on cell surfaces causing lysis (Fig 3).

Activation of the complement system is tightly controlled by a group of soluble and membrane‐bound regulators, including (i) C1‐inhibitor (C1INH) that inactivates C1r and C1s or MASP1 and MASP2 proteases and prevents CP‐ and MBL‐mediated complement activation, (ii) factors that accelerate the decay of convertases, such as the membrane‐bound CD55 or fluid phase factor H (CFH), and the transmembrane CD46, and (iii) CD59 that blocks the MAC assembly (Morgan & Wong *et* *al,* 1995; Zipfel & Skerka, 2009; Schmidt *et* *al,* 2016; Fig 3).

Although the main source of circulating complement components is the liver, complement proteins, receptors, and regulators are also produced and expressed locally by other cells, including immune cells and various tissue cells (Heeger & Kemper, 2012; Kolev, 2014). Locally produced complements are known to play an important role in tissue homeostasis, and thus, dysfunction or dysregulation of the system may contribute to various diseases. Recent studies have shown that the complement system can also be activated inside the cell, and intracellular complement activation is known to be involved in many important cellular functions, including intracellular pathogen recognition/elimination, cytokine production, and metabolism (Arbore *et* *al,* 2017; Kolev & Kemper, 2017; Liszewski *et* *al,* 2017).

### Complement regulation in the retina

As an immune privileged tissue, the retina is segregated from systemic circulation by various barriers (e.g., BRBs), and circulating complement proteins are not able to freely move into the retinal parenchyma under physiological conditions. However, the retina itself produces a variety of complement proteins, receptors, and regulators (Anderson *et* *al,* 2010). For example, transcripts of C1qb, C1r, C2, C3, C4, CFB, and CFH were detected in the retina and RPE/choroid of human (Anderson *et* *al,* 2010) and mouse eyes (Luo *et* *al,* 2011). Complement regulatory proteins such as CD46 (Vogt *et* *al,* 2006; Fett *et* *al,* 2012), CD55 and CD59 (Vogt *et* *al,* 2006), and CFH (Chen *et* *al,* 2007) as well as complement receptors CR1 and C3aR (Fett *et* *al,* 2012) and C5aR (Vogt *et* *al,* 2006) were found in retinal neurons and RPE cells.

Complement genes in the retina (Chen *et* *al,* 2010) and RPE/choroid (Chen *et* *al,* 2008) are expressed in an age‐dependent fashion. Also, the expression of C3, C4, and CFB in mouse retina can be affected by cataract surgery (Xu *et* *al,* 2011) and irradiation (Chen *et* *al,* 2012). Recently, an age‐related accumulation of MAC was found in the choriocapillaris of healthy donor eyes (Mullins *et* *al,* 2014; Chirco *et* *al,* 2016). The role of complement proteins in retinal cell homeostasis and activation remains to be fully elucidated, and this should be an important point of consideration when targeting the complement system for treating retinal diseases.

### The role of the complement system in retinal degeneration

The underlying pathologies of DR are retinal microvascular damage and neuronal degeneration. C3d and C5b‐9 have been detected in the choriocapillaris of DR eyes (Gerl *et* *al,* 2002) as well as in retinal vessels of patients suffering from type 2 diabetes for more than 9 years (Zhang *et* *al,* 2002), suggesting that the complement system may damage vascular endothelial cells through C5b‐9‐mediated lytic killing in diabetic eyes. In addition, uncontrolled complement activation may also contribute to pericyte loss in DR. Retinal pericyte‐reactive autoantibodies have been detected in patients with DR (Zhang *et* *al,* 2016) and *in vitro* studies have shown that the autoantibody‐initiated complement activation can induce pericyte damage and loss of function (Li *et* *al,* 2012). Increased C3a and C5a were detected in the serum (Zhang *et* *al,* 2016) and vitreous of patients with PDR (Muramatsu *et* *al,* 2013). C3a‐ and C5a‐induced inflammation may also contribute to DR pathogenesis. For example, Müller cells constitutively express C5aR and the expression can be upregulated by hyperglycemia and inflammatory stimuli such as prostaglandin E, which then results in the release of IL‐6 and VEGF, both known to be critically involved in DR pathology (Cheng *et* *al,* 2013).

The role of the complement system in the pathogenesis of AMD has been studied and reviewed extensively over the past decade (Warwick *et* *al,* 2014; Bora *et* *al,* 2015; McHarg *et* *al,* 2015). Key facts supporting the role of the complement system in the pathogenesis of AMD include the following: (i) Several complement components have been detected in drusen and AMD lesions (Anderson *et* *al,* 2002, 2010); (ii) higher plasma levels of C3a, C3d, Bb, and C5a have been observed in AMD patients (Scholl *et* *al,* 2008; Reynolds *et* *al,* 2009; Lechner *et* *al,* 2016); (iii) polymorphisms in a number of complement genes (CFH, CFB, C2, SERPING1, and C3) are genetic risk factors of AMD (Edwards, 2008; Cipriani *et* *al,* 2012); and (iv) inhibition of complement suppresses laser‐induced CNV in mice (Nozaki *et* *al,* 2006; Bora *et* *al,* 2010; Kim *et* *al,* 2013; Lipo *et* *al,* 2013). Mechanistically, CFH may inhibit CD47‐mediated resolution of subretinal inflammation and this inhibitory effect could be enhanced by the AMD associated CFH (H402) variant (Calippe *et* *al,* 2017).

A recent transcriptome study of two advanced stages of RP in dogs showed strongly increased gene expression of inflammasome and complement factors in the retina (Sudharsan *et* *al,* 2017). However, earlier studies reported reduced C3 and C4 levels and increased immune complexes in the sera from RP patients, and this reduced systemic complement activity appears to be related to poor disease prognosis (Heredia *et* *al,* 1984). The rhodopsin T17M mutation also reduces C3 secretion in RPE cells (Xiong *et* *al,* 2017), suggesting that some RP‐related genes may regulate complement expression/secretion by RPE cells. Humphries *et al* showed that C1q, the primary component of the classical pathway of the complement system, is a survival factor for cone cells, and C1q deficiency promoted photoreceptor death in Rho^−/−^ mice, a mouse model of Leber's congenital amaurosis (LCA; Humphries *et* *al,* 2012). Further understanding the role of complement system activation in RP may uncover novel targets for therapy.

### Modulating the complement system for the management of retinal degenerative diseases

Despite extensive research and significant advances in understanding the role of the complement system in retinal health and disease, the therapeutic value of these findings has only been tested in small groups of selected patients, such as the GA type of AMD (see below). A number of reasons may explain the slow progression in translations. First, complement dysregulation is not the primary cause of disease. The complement system only comes into play when retinal damage is evident. Therefore, modulating the complement system without addressing the initial cause of the disease may have limited impact on disease progression. Second, the physiological purpose of complement activation is likely to limit retinal damage and promote repair although excessive C3a, C5a, and C5b‐C9 may be detrimental. It is, therefore, critical to differentiate the beneficial roles from the detrimental roles of complement activation in retinal degeneration. Third, complement proteins and regulators may modulate retinal disease independent from the complement cascades. Complement‐based therapies are at different stages of clinical development for retinal diseases, particularly AMD (Xu & Chen, 2016).

Early clinical trials have proven that complement inhibitors are generally safe and well tolerated when injected intravitreally. The phase II/III studies have been conducted predominately in GA patients. For example, the latest C3‐targeted inhibitor APL‐2 has shown promising effect in the phase II study (NCT02503332), which reported a 29% reduction in the rate of GA lesion growth and 20% reduction in the group that received APL‐2 injection every other month. A greater effect was observed during the second 6 months of the study where a reduction in GA growth rate of 47 and 33% was seen in patients with monthly and every other month treatment, respectively. The phase III study is planned to begin in the second half of 2018 (Apellis‐Pharmaceuticals, 2018).

Lampalizumab (FCFD4514S) is a humanized IgG Fab fragment against CFD, thereby reducing the activation of the alternative complement pathway (Katschke *et* *al,* 2012). A phase II study (MAHALO) reported a 20% reduction in lesion area progression compared with sham control after 18‐month treatment of monthly intravitreal injections. Further subgroup analysis revealed a 44% reduction in patients who carry the CFI risk allele (rs17440077; Yaspan *et* *al,* 2017). However, further phase III studies (Spectri, NCT02247531; and Chroma, NCT02247479) failed to confirm the therapeutic effect in GA patients.

CLG561 is a fully humanized anti‐properdin antibody Fab fragment. Properdin promotes the association of C3b with CFB and provides a focal point for the assembly of C3 convertase C3bBb. A phase II study evaluating the safety and efficacy of intravitreal injections of CLG561 as a monotherapy and in combination with LFG316 in GA patients is ongoing (NCT02515942).

It must be noted that several other complement trials have ended early due to disappointing interim results. This highlights our lack of basic understanding of the mechanisms by which complement factors influence AMD. Thus, we are currently unable to address precisely when, where, and how to modulate the complement pathway in AMD and other retinal degenerative conditions.

## Targeting inflammasome activation in retinal degenerative diseases

### The inflammasome signaling pathway

The inflammasomes are cytosolic macromolecular signaling complexes that mediate IL‐1β and IL‐18 secretion the lytic cell death called pyroptosis. They play a crucial role in innate immunity by coordinating host immune response to invading pathogens or host‐derived danger signals. Assembly of inflammasomes is triggered by different PRRs, including nucleotide‐binding oligomerization domain‐like receptors (NLRs), absent in melanoma 2 (AIM2)‐like receptors (ALRs), or tripartite motif (TRIM) family receptors, which are capable of recognizing PAMPs and DAMPs. Assembly of the inflammasomes allows for the cleavage and activation of inflammatory caspases, which in turn cleave pro‐inflammatory cytokines pro‐IL‐1β and pro‐IL‐18 into their active forms.

Since NLRP1 was first described to form the inflammasome in 2002 (Martinon *et* *al,* 2002), members of the NLR family (NLRP1, NLRP3, and NLRC4) as well as other proteins (AIM2, pyrin) have been confirmed to initiate formation of inflammasomes (Broz & Dixit, 2016; Mathur *et* *al,* 2017). There are also other less well‐characterized PRRs, such as NLRP2, NLRP6, NLRP7, NLRP9b, NLRP12, IFN‐γ‐inducible protein 16 (IFI16), and retinoic acid‐inducible gene I (RIG‐I; also known as DDX58) which have also been reported to activate caspase‐1 (Broz & Monack, 2013; von Moltke *et* *al,* 2013; Broz & Dixit, 2016; Man & Kanneganti, 2016).

**Figure 4:**
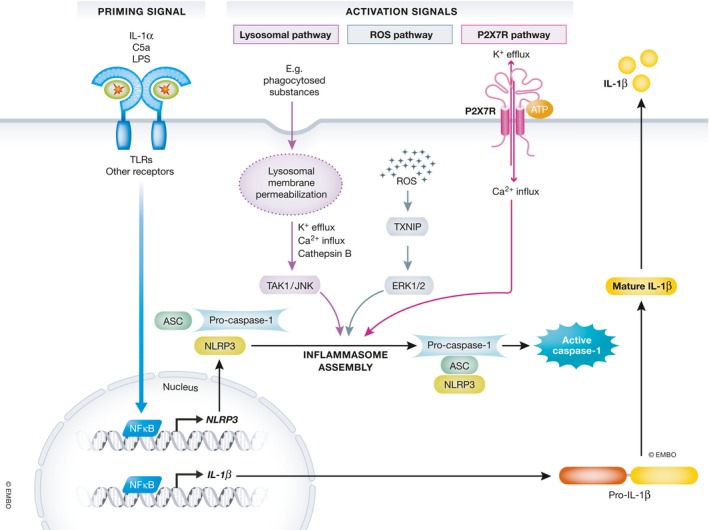
Molecular mechanisms of NLRP3 inflammasome priming and activation Schematic representation of the NLRP3 inflammasome pathway which requires two signals: (i) a priming signal which activates NF‐kB, subsequently promoting the transcription of NLRP3 and pro‐IL‐1β, and (ii) an activation signal which facilitates the oligomerization of NLRP3, ASC, and procaspase‐1, resulting in the activation of NLRP3 inflammasome and secretion of mature IL‐1β and IL‐18.

To date, the best studied and well‐characterized NLR molecule is NLRP3 (also known as NALP3, cryopyrin, CIAS1, and Pypaf1). Assembly of NLRP3 requires two signals: (i) a priming signal which activates NF‐kB, subsequently promoting the transcription of NLRP3 and pro‐IL‐1β, and (ii) an activation signal which facilitates the oligomerization of NLRP3, ASC, and procaspase‐1, resulting in the activation of NLRP3 inflammasome and secretion of mature IL‐1β and IL‐18 (Fig 4; Bauernfeind *et* *al,* 2009; Franchi *et* *al,* 2012, 2014; Juliana *et* *al,* 2012). In addition to the canonical inflammasomes, the non‐canonical inflammasome signaling pathways also exist, which target caspase‐11 in mice and caspase‐4 and caspase‐5 in humans. Shi *et* *al* (2014) have shown that caspase‐4/5/11 can directly respond to cytoplasmic LPS leading to self‐oligomerization and activation.

### Clinical data on involvement of inflammasome in retinal diseases

Aberrant inflammasome activation has been implicated in multiple diseases, including retinal diseases. For instance, Tarallo *et* *al* (2012) displayed that NLRP3, IL‐1β, and IL‐18 mRNA abundance in the RPE from human eyes with GA was markedly elevated compared to normal age‐matched control eyes. Others also observed upregulation of NLRP3, pro‐IL‐1β, and pro‐IL‐18 mRNA in the macula of both GA and nAMD (Cao *et* *al,* 2016; Wang *et* *al,* 2016b). Zhao *et* *al* (2015a) analyzed the protein level of pro‐IL‐1β and IL‐1β in vitreous samples from patients with retinal diseases. The results show that pro‐IL‐1β levels in nAMD, polypoidal choroidal vasculopathy (PCV), and Eales’ disease vitreous samples were significantly elevated, and IL‐1β expression in nAMD, PCV, Eales’ disease, and RVO vitreous samples was significantly elevated when compared with the control group. Interestingly, IL‐1β levels in serum samples of PCV and nAMD were significantly decreased in the same study.

Inflammation is assumed to be involved in the generation of neovascularization in PDR (Zhou *et* *al,* 2012). Most recently, Loukovaara *et* *al* (2017) reported that NLRP3 inflammasome activation is associated with the pathogenesis of PDR. It is also demonstrated that high intraocular pressure (IOP)‐induced retinal ischemia could trigger caspase‐8 signaling to activate NLRP1 and NLRP3 inflammasomes and IL‐1β secretion via TLR4 signaling in both mouse and rat models (Chi *et* *al,* 2014). These results provide new insights into the pathogenesis and development of new therapeutic strategies for clinical treatment by linking NLRP3 inflammasome and retinal diseases.

### Triggers of inflammasome activation in the retina

#### Oxidative stress

Increasing evidence suggests that ROS induces the activation of NLRP3 inflammasome and enhances the secretion of IL‐1β (Zhang *et* *al,* 2015; Choe & Kim, 2017). In STZ‐induced diabetic mice, rod demise was accompanied by an increase in LC3A protein, a marker for autophagosomes (Mizushima & Yoshimori, 2007; Piano *et* *al,* 2016). Similarly, ARPE‐19 cells showed signs of autophagy together with ROS release in response to high‐glucose‐induced stress (Shi *et* *al,* 2015). Inhibition of the autophagic response activated even more NLRP3 and caused IL‐1β release. The authors speculate that the cells incapable of removing ROS‐generating mitochondria may trigger NLRP3 inflammasome activation (Youle & Narendra, 2010; Shi *et* *al,* 2015). Thus, NLRP3 inflammasome activity is stimulated by ROS accumulation and counter‐balanced by autophagy (Zhou *et* *al,* 2010b).

Retinal lipofuscin contains lipid peroxidation‐ or glycoxidation‐induced end products 4‐hydroxynonenal (HNE), malondialdehyde (MDA), and advanced glycation end products (AGEs; Schutt *et* *al,* 2003). Kauppinen *et* *al* (2012) reported that HNE induced significantly increased NLRP3 mRNA levels and IL‐1β and IL‐18 production in RPE cells.

Thioredoxin (TRX)‐interacting protein (TXNIP), a TRX‐binding protein, is thought to be an endogenous inhibitor of TRX reductase activity. TXNIP dissociates from TRX at high concentrations of H_2_O_2_ and interacts with NLRP3. ROS‐dependent TXNIP–NLRP3 association was also found in monosodium urate crystals or R‐837‐treated macrophages (Zhou *et* *al,* 2010a). TXNIP can also mediate retinal inflammation, gliosis, and apoptosis in experimental diabetes (Devi *et* *al,* 2012). Zhou *et al* demonstrated a vital role of TXNIP in innate immunity through NLRP3 inflammasome activation and release of IL‐1β under oxidative stress. Recent studies demonstrated that ROS–TXNIP pathway mediates NLRP3 inflammasome activation in DR conditions *in vitro* and *in vivo* in rats. High glucose induces sustained upregulation of TXNIP, ROS generation, and inflammation in a Müller cell line of rats, and antioxidants or TXNIP silencing blocked IL‐1β and IL‐18 secretion in high‐glucose‐exposed human retinal microvascular endothelial cells (Devi *et* *al,* 2012; Chen *et* *al,* 2017). These results provide a potential therapeutic target for the treatment of DR.

#### Lysosomal membrane permeabilization

Lysosomal membrane permeabilization (LMP) is a key mechanism upstream of NLRP3 inflammasome activation, which induces subsequent cytosolic leakage of lysosomal components (Hornung *et* *al,* 2008; Stutz *et* *al,* 2009). Lysosome rupture triggers various cellular responses, including NLRP3 inflammasome activation, autophagy, and cell death (Okada *et* *al,* 2014). As lysosomes are organelles containing abundant amount of Ca^2+^, lysosome rupture induces Ca^2+^ influx from the lysosome into the cytosol activating the NLRP3 inflammasome through the CaMKII–TAK1–JNK pathway (Okada *et* *al,* 2014). TAK1 and JNK are activated in response to a soluble lysosomotropic agent L‐leucyl‐L‐leucine methyl ester (LLME) stimulus, and inhibitors of cathepsin B, cysteine proteases, or 5‐Z‐oxozeaenol (a TAK1 inhibitor) strongly attenuate the LLME‐induced activation of JNK. JNK is a family member of MAPK that responds to stress and that can regulate the activation of the NLPR3 inflammasome through ASC oligomerization. Furthermore, various LMP stimuli trigger significant K^+^ efflux (Munoz‐Planillo *et* *al,* 2013). Katsnelson *et* *al* (2015) reported that Ca^2+^ influx and K^+^ efflux are rapidly triggered after murine dendritic cell treatment with LLME. The lipofuscin component N‐retinylidene‐N‐retinyl‐ethanolamine (A2E) was also shown to trigger LMP (Taylor *et* *al,* 1992; Tomany *et* *al,* 2004). Brandstetter *et al* found that lipofuscin‐mediated phototoxicity results in LMP with cytosolic leakage of lysosomal enzymes and subsequent activation of caspase‐1 and inflammasome with secretion of IL‐1β and IL‐18 in RPE cells. NLRP3 inflammasome activation induced by LMP may contribute to AMD pathology through the release of pro‐inflammatory cytokines such as IL‐1β as well as through caspase‐1‐mediated pyroptosis (Tseng *et* *al,* 2013; Brandstetter *et* *al,* 2015b).

#### ATP and P2X7 receptor

P2X7R chiefly acts through the recruitment of the NLRP3 inflammasome complex (Giuliani *et* *al,* 2017). As a known and powerful activator of the NLRP3, P2X7R modulates NLRP3 expression at mRNA and protein levels, and excessive activation results in RPE cell death (Franceschini *et* *al,* 2015). During P2X7R opening, it directly allows K^+^ efflux and Ca^2+^ influx along the concentration gradient. K^+^ efflux is now acknowledged as a very potent stimulus for caspase‐1 activation and pro‐IL‐1β release that activates the NLRP3 inflammasome (Franchi *et* *al,* 2007; Petrilli *et* *al,* 2007; Franceschini *et* *al,* 2015). Recent evidences suggest that P2X7R and NLRP3 interact directly at discrete sub‐plasmalemmal cytoplasmic sites. P2X7R and NLRP3 can be co‐localized by confocal microscopy and co‐immunoprecipitated in both mouse microglia and mouse peritoneal macrophages (Franceschini *et* *al,* 2015).

Adenosine triphosphate promotes caspase‐1 activation, NLRP3 activation, IL‐1β and IL‐18 maturation and release, and cell death (Ferrari *et* *al,* 1997a; Perregaux *et* *al,* 2000). RPE cells and neural retina have been shown to release ATP in response to stimulation (Neal & Cunningham, 1994; Mitchell, 2001; Eldred *et* *al,* 2003; Newman, 2003; Pearson *et* *al,* 2005; Reigada & Mitchell, 2005; Reigada *et* *al,* 2005), which can act on P2X7R in the RPE cells via an autocrine or a paracrine manner (Perez *et* *al,* 1986; Xia *et* *al,* 2012). Yang *et* *al* (2011) reported that the P2X7R is expressed in both native and cultured human RPE cells and its activation induces both Ca^2+^ signaling and apoptosis in RPE cells. Furthermore, BzATP‐induced RPE apoptosis was blocked or significantly inhibited by P2X7R antagonists BBG, KN‐62, and oxidized ATP. Oxidized ATP, an irreversible blocker of P2X7R, abrogates ATP‐induced IL‐1β release from immune cells (Ferrari *et* *al,* 1997b). All of these above suggest that the over‐activation of P2X7R may contribute to the development of GA.

#### Complement components

Recently, Brandstetter *et* *al* (2015a) showed that complement component C5a is a priming signal for the NLRP3 inflammasome in RPE cells that mediates inflammasome activation by lipofuscin/blue light‐induced photo‐oxidative damage. There is also research showing that C1q represents an activation signal for the NLRP3 inflammasome, acting in a caspase‐1‐ and phagolysosome‐dependent manner in LPS‐primed mouse bone marrow‐derived macrophages and THP1 human monocytic cells (Doyle *et* *al,* 2012). In addition to C1q, C3a and MAC trigger inflammasome activation (Asgari *et* *al,* 2013; Triantafilou *et* *al,* 2013). C3a induces NLRP3 inflammasome activation and IL‐1β secretion in human monocytes by controlling the release of intracellular ATP into the extracellular space (Asgari *et* *al,* 2013). However, sublytic MAC attack generates pores on the membrane that allow Ca^2+^ influx, and thus increase cytosolic Ca^2+^ concentration, triggering NLRP3 activation and IL‐1β production (Triantafilou *et* *al,* 2013).

#### Amyloid‐β

Amyloid‐β (Aβ) is a component of drusen and has been suggested as pathogenic factor in AMD (Johnson *et* *al,* 2002). It is a pathogenic trigger peptide that induces inflammation and neurotoxicity in the retina. Intrinsic cytotoxicity of Aβ is due to its aggregated forms as soluble oligomers or insoluble fibrils (Gao *et* *al,* 2015). Aβ(1–40) and Aβ(1–42) are the two most common isoforms of Aβ, which are recognized to be the most relevant forms to induce neurodegeneration in amyloidosis (Zhang *et* *al,* 2012). Increasing Aβ(1–42) secretion was found in senescent ARPE‐19 cells (Glotin *et* *al,* 2008). Accumulating evidence suggests that increasing Aβ deposition with age may contribute to the development of AMD (Johnson *et* *al,* 2002; Dentchev *et* *al,* 2003; Zhao *et* *al,* 2015b). In addition to Aβ's cytotoxicity, NLRP3 inflammasome activation induced by Aβ may be responsible for RPE dysfunction. Halle *et* *al* (2008) reported that NLRP3 inflammasome activation is initiated by fibrillar Aβ‐induced lysosomal damage which increased release of lysosomal protease cathepsin B in microglia. As a trigger, Aβ stimulates RPE cells and results in accelerating the secretion of IL‐1β (Kurji *et* *al,* 2010).

#### Alu RNA

Alu RNA, a non‐coding RNA transcribed from Alu elements, plays a prominent role as gene modulator via genome shaping, transcriptional regulation, and mRNA alternative splicing (Hasler *et* *al,* 2007). Alu RNA accumulation secondary to DICER1 deficiency in the RPE has been implicated in GA (Tarallo *et* *al,* 2012). Kaneko *et* *al* (2011) showed that a reduction in RNase DICER1 leads to accumulation of Alu RNA transcripts in the RPE of GA patients. Delivery of a plasmid coding for Alu RNA upregulated NLRP3 and IL‐18 mRNAs in mouse RPE cells and induced ROS production in human RPE cells (Tarallo *et* *al,* 2012). These results suggest that Alu RNA triggers NLRP3 priming and mitochondrial ROS in RPE cells. ERKs promote cell death in a variety of chronic neurodegenerative states. Increased ERK1/2 phosphorylation was observed in the RPE of human eyes with GA, and Alu RNA‐induced RPE degeneration in mice is rescued by intravitreous administration of PD98059, an inhibitor of the ERK1/2‐activating kinase MEK1 (Dridi *et* *al,* 2012). Thus, RPE degeneration induced by DICER1 depletion or Alu RNA over‐expression may be mediated by ERK1/2 signaling (Dridi *et* *al,* 2012). ERK1/2 signaling also regulates angiogenesis and CNV (Hua *et* *al,* 2011; Xie *et* *al,* 2011). Therefore, ERK1/2 activation is a potential target for both atrophic and neovascular AMD.

### Strategies for therapeutic modulation of inflammasome activation

#### IL‐1β inhibitors

IL‐1β is a key inflammatory cytokine regulated by the inflammasome, and increased levels of IL‐1β are present in DR (Kowluru *et* *al,* 2011; Liu *et* *al,* 2012) and AMD (Lavalette *et* *al,* 2011; Tarallo *et* *al,* 2012). Amelioration of IL‐1β activation prevents mitochondrial dysfunction and DNA damage (Kowluru *et* *al,* 2011). Furthermore, glucose‐induced apoptosis of retinal endothelial cells is prevented by neutralization of IL‐1β through incubating the cells with an IL‐1β antibody or IL‐1β receptor antagonist (Kowluru & Odenbach, 2004). The importance of IL‐1β in retinal diseases makes IL‐1 inhibition a therapeutic option. The IL‐1β receptor antagonist anakinra (ANA), anti‐IL‐1β antibody canakinumab (CAN), and recombinant humanized anti‐IL‐1β antibody gevokizumab (XOMA 052) have good clinical results in ocular diseases such as uveitis secondary to Behçet's disease (BD; Gul *et* *al,* 2012; Ugurlu *et* *al,* 2012; Vitale *et* *al,* 2014; Cantarini *et* *al,* 2015; Emmi *et* *al,* 2016). ANA and CAN have been shown to be an effective and safe therapeutic option for BD‐related refractory or long‐standing uveitis with a significant reduction in the rate of ocular inflammatory flare, resolution of active retinal vasculitis, preservation of visual acuity, and significant decrease in required steroid dosages (Fabiani *et* *al,* 2017). Ildefonso proposed that anti‐inflammatory genes delivered by an adeno‐associated virus (AAV) vector could be used as potential treatments for retinal inflammation (Ildefonso *et* *al,* 2015). Eyes injected with the caspase activation and recruitment domain (CARD) AAV vector had a significant decrease in both IL‐1β secretion and infiltrating cells (Ildefonso *et* *al,* 2015). Data on the efficacy of IL‐1β inhibition therapy in inflammasome‐related retinal diseases are currently lacking.

#### NLRP3 inhibitors

Direct inhibition of NLRP3 is an obvious approach for suppressing inflammasome activity. Recently, a small‐molecule inhibitor MCC950 (also known as CRID3), which is a diarylsulfonylurea‐based compound, was reported to be a potent and highly specific inhibitor of NLRP3, but not the AIM2, NLRC4, and NLRP1 inflammasomes (Coll *et* *al,* 2015). Coll *et* *al* (2015) reported that MCC950 could suppress both canonical and non‐canonical NLRP3 activation by preventing ASC complexes instead of blocking K^+^ efflux, Ca^2+^ flux, or NLRP3–ASC interactions. By reducing IL‐1β and IL‐18 secretion, the substance alleviated the severity of EAE and cryopyrin‐associated periodic syndromes (CAPS) in mouse models. MCC950 thus is a potential therapeutic for NLRP3‐associated diseases. It has also been reported in APP/PS1 mice, an AD model, that MCC950 suppresses inflammasome activation and IL‐1β production, stimulates Aβ phagocytosis *in vitro*, and reduces Aβ accumulation (Dempsey *et* *al,* 2017). With regard to the retina, MCC950 is capable of inhibiting NLRP3 inflammasome activation and apoptosis in human retinal endothelial cells (HRECs) under high‐glucose conditions, likely through downregulation of the Nek7–NLRP3 pathway (Zhang *et* *al,* 2017).

Another substance that inhibits NLRP3 is the ketone body β‐hydroxybutyrate (BHB; Youm *et* *al,* 2015). Unlike MCC950, BHB blocks only the canonical NLRP3 inflammasome activation pathway by preventing K^+^ efflux and reducing ASC oligomerization and speck formation (Youm *et* *al,* 2015). It reduces IL‐1β and IL‐18 production in human monocytes and attenuates caspase‐1 activation and IL‐1β secretion in mouse models of NLRP3‐associated diseases, such as Muckle–Wells syndrome, familial cold autoinflammatory syndrome, and urate crystal‐induced peritonitis. Both the MCC950 and BHB hold promise as potential novel pharmaceutical approach for treating DR, AMD, and other NLRP3‐induced ocular diseases.

#### Nucleoside reverse transcriptase inhibitors

Nucleoside reverse transcriptase inhibitors (NRTIs) are widely used to treat AIDS by blocking HIV replication. Fowler *et* *al* (2014) discovered that NRTIs inhibit P2X7‐mediated NLRP3 inflammasome activation independent of reverse transcriptase inhibition. Clinically relevant NRTIs such as lamivudine (3TC), stavudine (d4T), and abacavir (ABC) were shown to block caspase‐1 activation induced by Alu RNA in RPE cells (Fowler *et* *al,* 2014). Furthermore, NRTIs were efficacious in mouse models of GA and choroidal neovascularization. Intravitreous injection of the NRTIs 3TC, zidovudine (AZT), and ABC significantly suppressed laser‐induced CNV and VEGF‐A secretion in wild‐type mice but not P2rx7^−/−^ mice (Mizutani *et* *al,* 2015). This suggests NRTIs as a possible new therapeutic approach for both dry and wet AMD.

## Conclusion

In the current review, we point out the fundamental similarity between AMD, DR, and hereditary retinopathies (using the example of RP)—and possible other retinal diseases as well—namely non‐resolving and overwhelming inflammation. We pointed out three arms of inflammation which are activation of (i) mononuclear phagocytes, (ii) the complement system, and (iii) the inflammasome. All three arms are intertwined and cannot be treated exclusively without affecting each other, hence representing an attractive therapy target. Complement receptors are expressed on MPs while NLRP inflammasomes lead to IL‐1β activation and secretion contributing significantly to microglial activation and macrophage recruitment. Perturbations of immune‐suppressive capacities of the RPE, retinal neurons, and macroglia due to aging, hyperglycemia, or other defects evoked by genetic risk variants lead to non‐resolving inflammation. Hence, the proposed therapeutic options are auspicious approaches to start with.

**Pending issues**

(i) Experimental studies on separating retinal immune cell populations.
(ii) Linking retinal microglia and macrophage phenotypes and functions with disease outcome.
(iii) Identifying optimal targets in the complement cascade.
(iv) Clinical studies with immunomodulatory compounds.

## Conflict of interest

T.L. is named inventor on a patent application related to the use of polysialic acid for neurodegenerative diseases filed by the universities of Bonn and Cologne. All other authors declare that they have no conflict of interest.

## For more information

(i) http://expimmeye.uni-koeln.de/
(ii) http://www.vision-research.eu/
(iii) http://www.pro-retina.de/
(iv) http://www.for2240.de/
